# Comparative analysis of *Penicillium* genomes reveals the absence of a specific genetic basis for biocontrol in *Penicillium rubens* strain 212

**DOI:** 10.3389/fmicb.2022.1075327

**Published:** 2023-01-13

**Authors:** Elena Requena, Lola Alonso-Guirado, Javier Veloso, María Villarino, Paloma Melgarejo, Eduardo Antonio Espeso, Inmaculada Larena

**Affiliations:** ^1^Grupo Hongos Fitopatógenos, Departamento de Protección Vegetal, Instituto Nacional de Investigación y Tecnología Agraria y Alimentaria, Consejo Superior de Investigaciones Científicas (INIA-CSIC), Madrid, Spain; ^2^Grupo de Epidemiología Genética y Molecular, Centro Nacional de Investigaciones Oncológicas (CNIO), Madrid, Spain; ^3^Departamento de Biología Funcional, Escuela Politécnica Superior de Ingeniería, Universidad de Santiago de Compostela, Lugo, Spain; ^4^Laboratorio de Biología Celular de Aspergillus, Departamento de Biología Celular y Molecular, Centro de Investigaciones Biológicas Margarita Salas, CSIC (CIB-CSIC), Madrid, Spain

**Keywords:** PO212, biocontrol agent, *Penicillium rubens*, genome, comparative genomics

## Abstract

*Penicillium rubens* strain 212 (PO212) is a filamentous fungus belonging to the division Ascomycete. PO212 acts as an effective biocontrol agent against several pathogens in a variety of horticultural crops including *Fusarium oxysporum* f.sp. *lycopersici*, causing vascular wilt disease in tomato plants. We assembled draft genomes of two P. rubens strains, the biocontrol agent PO212 and the soil isolate S27, which lacks biocontrol activity. We also performed comparative analyses of the genomic sequence of PO212 with that of the other *P. rubens* and *P. chrysogenum* strains. This is the first *Penicillium* strain with biocontrol activity whose genome has been sequenced and compared. PO212 genome size is 2,982 Mb, which is currently organized into 65 scaffolds and a total of 10,164 predicted Open Reading Frames (ORFs). Sequencing confirmed that PO212 belongs to *P. rubens* clade. The comparative analysis of the PO212 genome with the genomes of other *P. rubens* and *Penicillium chrysogenum* strains available in databases showed strong conservation among genomes, but a correlation was not found between these genomic data and the biocontrol phenotype displayed by PO212. Finally, the comparative analysis between PO212 and S27 genomes showed high sequence conservation and a low number of variations mainly located in ORF regions. These differences found in coding regions between PO212 and S27 genomes can explain neither the biocontrol activity of PO212 nor the absence of such activity in S27, opening a possible avenue toward transcriptomic and epigenetic studies that may shed light on this mechanism for fighting plant diseases caused by fungal pathogens. The genome sequences described in this study provide a useful novel resource for future research into the biology, ecology, and evolution of biological control agents.

## Introduction

*Penicillium rubens* strain 212 (PO212, ATCC201888), formerly known *Penicillium oxalicum* (Villarino et al., 2016), is a filamentous fungus belonging to the division Ascomycete. PO212 is a strain that mainly attracts agricultural and biotechnological interest because it is an effective biocontrol agent (BCA) against several pathogens in a variety of horticultural crops (Larena et al., 2003b; De Cal et al., 2008, 2009; Martinez-Beringola et al., 2013). Among the fungal pathogens, PO212 acts against *Fusarium oxysporum* f.sp. *lycopersici* (FOL; Sacc.) W. C. Snyder and H. N. Hans, causing vascular wilt disease in tomato plants (De Cal et al., 1995). The effective control of tomato wilt is based on the application of PO212 conidia (Pascual et al., 2000) and conidial contact with roots (De Cal et al., 2000). Plant–fungus contact is achieved by watering seedlings 7 days before transplanting, in seedbeds with a conidial suspension of PO212, at a final conidial density in the seedbed substrate and rhizosphere between 10^6^ and 10^7^ conidia per gram (De Cal et al., 1999, 2000; Larena et al., 2003b). PO212 acts against Fusarium wilt primarily through a mechanism of induced resistance in tomato plants (De Cal et al., 1997b, 1999, 2000). Nevertheless, a previous study showed that the competition for space and nutrients (Sabuquillo et al., 2009) and the promotion of plant growth have been demonstrated (De Cal et al., 1995).

Genome sequencing is currently on the rise due to new and faster procedures and reduced sequencing costs. This allows for expanded comparative studies of the genomes of organisms with key roles in health, biotechnology, or agriculture. Among these interesting organisms are species of the genus *Penicillium*, especially *Penicillium chrysogenum* and *P. rubens*. However, controversy remains about the true existence of these two separate species (Houbraken et al., 2011). Genome sequences of several *P. chrysogenum* and/or *P. rubens* strains have been reported. In genome databases, the following strains are classified as *P. chrysogenum*: P2niaD18 (Specht et al., 2014), HKF42 (Gujar et al., 2018), KF-25 (Peng et al., 2014), NCPC10086 (PENC1.0 0; Wang et al., 2014), and v1.0 (de Vries et al., 2017). Meanwhile, the strains PrWis (*P. rubens* Wisconsin 54-1255; Van Den Berg et al., 2008) and Biourge 1923 (IMI 15378; Pathak et al., 2020) are classified as *P. rubens*. PrWis was the first strain of *P. rubens* to be sequenced (Van Den Berg et al., 2008). The strains P2niaD18 and PrWis were interesting from a sanitary point of view because of their high penicillin-producing capacity. Notably, these two strains are descendants from the natural isolate NRRL 1951 which was classified as *P. chrysogenum* (Martín, 2020). Both strains have undergone various mutagenesis procedures and screening methods to improve their respective penicillin yields. This implies that the presence of sequence modifications and reorganizations in the genomes of these strains is highly expected.

*Penicillium rubens* strain 212 was initially misclassified as *P. oxalicum* based on morphological characteristics, such as the color of the spore layer on the colony surface, the size and shape of the colony, conidial size, and conidiophore morphology (Ramírez, 1982). Subsequent sequencing of internal transcribed spacers (ITS) regions and the isolation and identification of 5-fluoroorotic acid (5-FOA)-resistant mutants showed that PO212 was a *P. rubens* strain (Villarino et al., 2016). The first study to understand the genetic basis of PO212 biocontrol activity (BA) focused on the analysis of genes involved in nitrate assimilation since PO212, and other *P. rubens* strains showing a BA against Fusarium wilt lacked the ability to use nitrate as the main nitrogen source (Espeso et al., 2019). This nitrate assimilation-deficient phenotype was due to the presence of mutations in the NirA regulator or the nitrate transporter CrnA. However, the complementation of these mutations did not help to understand the BA of PO212 (Espeso et al., 2019).

Genome-wide analyses proved to be a good method to determine the genetic basis behind the biocontrol process in several organisms (Pattemore et al., 2014; Sharma et al., 2017; Piombo et al., 2018). Moreover, comparative genomics allowed the identification of pathways or mutations between the genomes of different organisms that may be specific to organisms with BA (Massart et al., 2015). Comparative analyses of *Trichoderma* spp. genomes revealed notable differences in contrast to the genomes of other multicellular ascomycetes in comparison to publicly available genomes (Kubicek et al., 2011). These analyses of *Trichoderma* spp. represent a useful new resource for the further development of improved and research-driven strategies to select and improve *Trichoderma* species as BCA (Kubicek et al., 2011). However, the comparative genomics of two biocontrol strains of *Metschnikowia fructicola* revealed a very high mutation rate, which may suggest that *M. fructicola* could undergo genomic changes to adapt to plant surfaces, tolerate a variety of environmental stresses, and survive under nutritional restrictions (Piombo et al., 2018). Major efforts in technology and bioinformatics tools have significantly increased our knowledge of BCA and their properties (Massart et al., 2015).

In this study, we conducted a comparative genomic approach to understand the genetic basis of BA in *P. rubens*. We assembled draft genomes of two *P. rubens* strains, the BCA PO212 and the soil isolate S27, which lacks BA. We also performed comparative analyses of the genomic sequence of PO212 with that of the other *P. rubens* and *P. chrysogenum* strains. These analyses revealed significant conservation of genomic sequences among all strains compared and evidenced the absence of any specific genes related to biocontrol in PO212.

## Materials and methods

### Strains and growth conditions

*Penicillium rubens* strain 212 and other *P. rubens* strains, isolated from diverse agricultural soils and plant samples in Spain, are listed in Table 1. Conidia from these strains were stored in 20% glycerol for a long term at −20°C except for PO212, which was stored at 4°C as dried conidia. Dried conidia of PO212 were produced in a solid-state fermentation system and dried as previously described by Larena et al. (2003a). *Penicillium* strains were grown on potato dextrose agar (PDA) or minimal medium (MM; Espeso et al., 2019) with 5 mM ammonium tartrate and D-glucose 1% (w/v) as nitrogen and carbon source, respectively, and incubated at 25°C for 5 days. For short-term storage, strains were kept at 4°C on solid media.

**Table 1:** List of *Penicillium rubens* strains used in this work.

Strain	Origin	Host	BCA^a^	References
PO212	Spain	Soil	+	De Cal et al. (1995)
S27	Spain (Ávila)	Soil	–	Villarino et al. (2016)
S17	Spain (Segovia)	Soil	–	Villarino et al. (2016)
S71	Spain (Segovia)	Soil	–	Villarino et al. (2016)
S73	Spain (Segovia)	Soil	+	Villarino et al. (2016)
CH2	Spain (Madrid)	Leaf of a perennial plant	–^b^	This work
CH5	Spain (Madrid)	Shoot of a perennial plant in a field of peach trees	–^b^	This work
CH6	Spain (Madrid)	A deep soil sample in the field of peach trees	+^b^	This work
CH8	Spain (Madrid)	A shoot of a perennial plant in a pine forest	+^b^	Espeso et al. (2019)
CH16	Spain (Lérida)	Outbreak of pruning	+^b^	This work

^a^BCA: Biological Control Agent.

^b^Unpublished results, general screenings of BCA strains INIA-CSIC. The symbol + indicates biocontrol activity. The symbol – indicates no biocontrol activity.

The biocontrol activity of *P. rubens* strains was tested using the pathogenic isolate 1A of FOL, provided by Dr. Cristina Moyano from the Laboratory for Assessment of Variety, Seed and Nursery Plants, INIA-CSIC (Madrid, Spain). FOL was stored at 4°C in tubes containing sterile sand. For mycelial production, conidia from FOL stored in sterile sand were germinated on Czapek Dox agar (CDA; Difco Laboratories, Detroit, MI, United States) and cultivated in darkness at 25°C for 7 days. Microconidial inoculum of FOL was produced in 250 ml flasks containing 150 ml of sterile Czapek Dox broth (Difco). Each flask was inoculated with three mycelial plugs (1 cm diameter) from the 7-day-old cultures on CDA (De Cal et al., 1995) and incubated for 5 days at 25°C in a rotary shaker (model 3527; Lab-Line Instruments, Inc.) at 150 rpm. Microconidia were separated from the mycelial mass by filtration through glass wool. The conidial concentration was determined using a hematocytometer and adjusted to 10^6^ microconidia/ml.

### Efficacy assays

At least two growth chamber experiments were carried out on tomato plants to evaluate the biocontrol efficacy of S27 against FOL as described by Villarino et al. (2018). Seeds of tomato cultivar “San Pedro”, which is susceptible to races 1 and 2 of FOL, were used in all experiments. Tomato seeds were sown in sterile trays (27 cm × 42 cm × 7 cm) that contained an autoclaved (for 1 h at pressure of 1 kg cm^−2^ and temperature of 121°C, during 3 consecutive days) mixture of vermiculite (Termita; Asfaltex, S.A., Barcelona, Spain) and peat (Gebr. BRILL substrate GmbH & Co. KG; 1:1, *v:v*). The trays were maintained in a growth chamber at 25°C with fluorescent lighting (100 μE m^−2^ s^−1^, 16-h photoperiod) and 80–100% relative humidity for 3–4 weeks. Tomato seedlings (with at least two true leaves) were treated 7 days before transplanting with an aqueous conidial suspension (6 × 10^6^ conidia per gram of substrate) of S27 or PO212. Conidial suspensions of PO212 and S27 were prepared as follows: Dried conidia of PO212 were rehydrated in sterile distilled water (SDW) using a rotatory shaker at 150 rpm for 2 h (CERTOMAT® RM). Conidia of S27 were harvested from colonies grown on PDA and incubated in the dark at 25°C for 7 days. The day before treatment, the viability of PO212 and S27 conidia were estimated by measuring their germination as previously described (Larena et al., 2003a). For each replicate (three by sample type), the germination of 50 randomly selected conidia was counted and viability was calculated and expressed as a percentage (De Cal et al., 1990). Seven days after treatment, tomato seedlings were transplanted from seedbeds into 100-ml flasks containing 100 ml of Hoagland solution (Hoagland and Arnon, 1950) so that the roots were in contact with the solution, as described by De Cal et al. (1997a). An aliquot of an aqueous (SDW) conidial suspension of FOL was added to the flasks just before transplanting so that the final conidial concentration in the flasks was 1 × 10^5^ conidia/ml. Plants that had been inoculated with the pathogen but not treated with any strain of *P. rubens*, were used as the control. Five replicate flasks, each containing four plants, were used per treatment. The flasks were placed in a randomized complete block design in a growth chamber for 4 weeks under the conditions described earlier in this subsection. The complete experiment was done two times. Disease severity was graded on days 7, 14, 21, and 28 after the transplant. Disease severity followed a 1–5 index scale: 1, healthy plants (0–24%); 2, yellow lower leaves (25–49%); 3, dead lower leaves and some yellow upper leaves (50–74%); 4, dead lower leaves and wilted upper leaves (75–99%); and 5, dead plants (100%; De Cal et al., 1995). All plant roots were placed in humidity chambers at the end of each experiment, and the presence or absence of the pathogen in the crown after 5 days of incubation at 25°C was recorded.

Data of disease severity and incidence were analyzed by ANOVA with the STATGRAPHICS program (XVII Centurion. v. 17.2.00). When the *F* test was significant at a value of *p* of < 0.05, means were compared using the Student–Newman–Keul’s multiple range test (Snedecor and Cochran, 1980).

### Genomic DNA extraction, sequencing, and PCR

Genomic DNA (gDNA) was extracted from the mycelia of *Penicillium* strains (Table 1) grown in liquid MM at 25°C for 2 days. Mycelia were harvested by filtration using Miracloth (Calbiochem, Merck-Millipore, Darmstadt, Germany). Samples were lyophilized for at least 6 h. For each sample, mycelium was pulverized using a ceramic bead in a FastPrep-24 homogenizer (MP Biomedicals™), one pulse for 20 s at minimum speed. A sample of 100 mg of powdered mycelium was mixed with 1 ml of DNA extraction solution (25 mM Tris–HCl pH 8.0, 250 mM sucrose, and 20 mM EDTA pH 8.0). Before incubation for 15 min at 65°C, 100 μl of 10% SDS were added to each sample. Next, proteins and cellular debris were removed by adding 1 ml of Phenol/Chloroform/Isoamyl alcohol mixture per sample and further mixing on a rotary shaker (Rotator Multi Bio RS-24) for 15 min at room temperature (RT). Organic and aqueous phases were separated by centrifugation on a benchtop centrifuge at maximum speed for 5 min. gDNA was precipitated from the aqueous phase by the addition of 1/10 vol of sodium acetate pH 6 and 0.6 vol of 2-propanol, followed by incubation for 15 min at RT and centrifugation for 5 min at RT. To wash the gDNA pellet, 1 ml ethanol (80%) was added, followed by centrifugation at max. Speed for 5 min at RT. After drying the ethanol in samples, pellets were dissolved in 500 μl sterile water, and samples were treated with DNase-free RNase A (5 mg/ml; 37°C, 60 min). gDNA was newly precipitated and washed as described before. Finally, gDNA was dissolved in 200 μl nuclease-free water and stored at −20°C until use.

Sequencing of PO212 gDNA was performed on an Illumina MiSeq 500 cycles at the “FPCM, Fundación Parque Científico de Madrid” sequencing facility using 150 bp and 250 bp paired-end sequencing reads. For S27 gDNA, Stab Vida (Portugal) performed the construction and sequencing of DNA libraries. DNA libraries were sequenced on the lllumina Hiseq 4000 platform, using 150 bp paired-end sequencing reads.

Standard PCR protocols were used: initial denaturation at 95°C for 2 min, 30 cycles of denaturation at 95°C for 10 s, annealing at 55°C for 30 s, and extension at 72°C for 1 min/kb. After 30 cycles, an extension at 72°C for 10 min and storage at 4°C. The polymerase Takara (TaKaRa Taq™) was used for amplification. Oligonucleotides were designed using Vector NTI™ Suite 8 and are listed in Table 2. The oligonucleotides for the amplification of MAT1-1 and MAT1-2 were previously described in Espeso et al. (2019). PCR products were analyzed in 0.8% agarose-TAE electrophoresis and when required, they were purified using the PCR clean-Up kit (Macherey-Nagel), following the manufacturer’s instructions. Sequencing of DNA fragments was done by Stab Vida’s (Portugal) sequencing service.

**Table 2:** List and nucleotide sequence of the oligonucleotides used in this work.

Primer code	5′-3′ Sequence
g196 F	GGACAGTACGGCATTGGATATTACGGACACC
g196 R	CCAGATTGTGTCCCATAGACGTTGTCCG
g1339 F	CCACACCGTCAGACTTTGGAATCCTATACC
g1339 R	CGACTTGCACGACAAGATGAGTTGGTTTCC
g3160 F	GCTCCGCTGGGAATGTATTATACACCTACG
g3160 R	CTCGCAATTCCTCTTGAGATGGAAGCTCG
g3741 F	GGATCGAACACGAGGGAAGATTCCTTGCC
g3741 R	CCAACACTGTTACAGAAAGCCTCGATGG
g3975 F	GCCAAAGCTCAACCAATACCCAGAGTACC
g3975 R	CGACTATGTCGCTAATTCGCAGGGTCGTGTC
g4471 F	CCAAGATCACCTCAACTTGTCTGCCTCACC
g4471 R	CGTATGGAGGAGCAACGATGAAAGAGGATCG
MAT1-1_fw	TGCAGCTCAAGTTCTACG
MAT1-1_rv^a^	AGGAGTACATCTCATCAACC
MAT1-2_fw	ATGGTGAAGTCTTCCTGCC
MAT1-2_rv^a^	AGAGAGTGGCTCGACACC
nirA 1	CACTAGGCATGCGAAGAGG
nirA 2	TACATCGCTGCTGATCTCGC

a These sequences correct the sequences previously published in Espeso et al. (2019).

### Assemblies and comparative analyses of genomes

For sequencing of the PO212 genome, two libraries of 150 and 250 bp paired-end fragments were produced and raw sequencing reads were subjected to quality control using the FastQC program.^1^ A Q > 28 along the read length and the *k*-mers and nucleotide distribution were homogeneous in both libraries. An A5-miseq pipeline was used (Coil et al., 2015) to assemble the PO212 genome. The nuclear reads were extended using FLASH (Magoč and Salzberg, 2011) and were assembled using the A5-miseq pipeline, until the output, named final.scaffolds.fasta, was obtained. The previously aligned mitochondrial reads were assembled to obtain a single mitochondrial contig of ~28 kbp.

*De novo* assembly of the S27 genome was performed using the A5-miseq pipeline (Coil et al., 2015) and further revised with Gapcloser from the Soapdenovo assembler (Luo et al., 2015). The aligned S27 mitochondrial reads were assembled apart from the genome in 13 contigs of ~34 kbp.

Completeness of the genome assemblies was assessed by the Benchmarking Universal Single-Copy Orthologs (BUSCO) v.2.0.1 software tool (Simão et al., 2015). The assembled genomes were annotated using the MAKER (v.2.31.9) pipeline (Campbell et al., 2014). Before annotation, a species-specific repeat library was constructed using RepeatModeler (v.1.0.8) to mask repeats (Tempel, 2012). Gene models were predicted with AUGUSTUS (Stanke et al., 2006) using two strategies, *ab initio* gene predictors and training with *Aspergillus nidulans* as a model genome.

Gene models of the PO212 and S27 genomes were manually curated using the P2niaD18 (GCA_000710275) and PrWis (GCA_000226395) strain proteins available in the EnsemblFungi and the National Center of Biotechnology Information (NCBI) database as evidence for gene prediction using Apollo (Dunn et al., 2019). Predicted proteins were functionally annotated using BLASTp (Altschul et al., 1990) against the non-redundant database of the NCBI and classified using InterProScan and Pfam analysis (Jones et al., 2014).

The comparison of the PO212 genome with genomes from other strains of *P. rubens* and *P. chrysogenum*, which are available in the database and listed in Table 3, was performed through the Quast bioinformatics tool (Gurevich et al., 2013). To visualize the genomes of the *P. rubens* and *P. chrysogenum* strains, Icarus, a genome visualizer based on the Quast tool, was used (Mikheenko et al., 2016). These genomes are divided into two groups according to the literature: *P. chrysogenum* (P2niaD18, KF-25, HKF42, NCPC10086, and v1.0) and *P. rubens* (PrWis and Biourge 1923; Table 3).

**Table 3:** List of fungal strains whose genomes have been used in this work.

Strain	Species	Assembly	References
PO212	*Penicillium rubens*	JAPDLE000000000	This work
S27	*P. rubens*	JAPDLD000000000	This work
PrWis	*P. rubens*	GCA_000226395.1	Van Den Berg et al. (2008)
Biourge 1923	*P. rubens*	GCA_902636305.1	Pathak et al. (2020)
P2niaD18	*Penicillium chrysogenum*	GCA_000710275.1	Specht et al. (2014)
NCPC10086	*P. chrysogenum*	GCA_000523475.1	Wang et al. (2014)
HKF42	*P. chrysogenum*	GCA_002080375.1	Gujar et al. (2018)
KF-25	*P. chrysogenum*	GCA_000816005.1	Peng et al. (2014)
v1.0	*P. chrysogenum*	JGI Mycocosm	de Vries et al. (2017)
HP7-1	*Penicillium oxalicum*	GCA_001723175.3	Zhao et al. (2016)
FGSC-A4	*Aspergillus nidulans*	GCA_000011425.1	Wortman et al. (2009)

Due to the current controversy in the classification of *P. chrysogenum* and *P. rubens*, in this study, all these strains have been classified as belonging to the *P. chrysogenum or P. rubens* clade, based on the barcodes of three genes encoding proteins: β-tubulin (BenA), RNA polymerase II second largest subunit (RPB2), and calmodulin (CaM). The barcodes used were JF909949 (BenA), JX996658 (RPB2), and JX996263 (CaM) to *P. rubens* and AY495981 (BenA), JN121487 (RPB2), and JX996273 (CaM) to *P. chrysogenum* (Visagie et al., 2014). The sequences of *P. rubens* and *P. chrysogenum* were aligned to the barcodes with the Multiple Sequence Alignment tool of Clustal Omega (EMBL-EBI).

MUMmer4 software package (Marçais et al., 2018) was used for pairwise alignment of PO212 and S27 assemblies by setting PO212 as the reference; the minimum length of a matched group to 20 bp and the distance an alignment extension will attempt to extend to poor scoring regions before yielding to 100 bp. Comparative analysis was performed using Circos tools (Krzywinski et al., 2009). Moreover, CLC Genomics Workbench 12 (QIAGEN Bioinformatics; https://digitalinsights.qiagen.com/products-overview/discovery-insightsportfolio/analysis-and-visualization/qiagen-clc-genomics-workbench/) was used to align the reads of S27 to the assembled genome of PO212. The CLC alignment workflow was used to map the raw reads to a reference genome and to detect variants. Default values were used, except for the minimum coverage and minimum count, which were set to 10 and 2, respectively, to avoid loss of information. Of all the variations found, homozygous variations were selected. Only variations with 100% of frequency were taken into account for manual verification by PCR amplification using specific primers and sequencing. To visualize amino acid changes in genes between PO212 and S27 strains, we used Integrative Genome Viewer (IGV version 2.9.4; Robinson et al., 2011).

### Phylogenetic analysis

A phylogenetic tree was constructed based on nucleotide sequence from assemblies of genomes from PO212, S27, and other strains of *P. rubens* and *P. chrysogenum* available in the database and listed in Table 3. For this purpose, *Aspergillus nidulans* strain FGSC-A4 genome (Wortman et al., 2009) and *P. oxalicum* strain HP7-1 genome (Zhao et al., 2016) were used as outgroups. The phylogenetic tree was generated using the software JolyTree (Criscuolo, 2019). The algorithm uses an alignment-free distance-based procedure for inferring phylogenetic trees from genome contig sequences. The pairwise dissimilarity between each pair of genomes was estimated using the Mash method (Ondov et al., 2016). Balanced Minimum-Evolution (BME) was used to optimize the evolutionary distances calculated in the first step. Finally, the rate of elementary quartets (REQ) was used for assessing the branch confidence values to construct the final tree (Criscuolo, 2019).

In addition, a tree obtained with the concatenated sequences *benA, caM*, and *RPB2* barcodes was generated using the maximum likelihood method with 1,000 bootstrap replications. The tree was drawn to scale, with branch lengths representing the inferred evolutionary distances. Between 1,879 and 1,931 bp were presented in the final data set. Sequences of *P. oxalicum* barcodes were used as an outgroup. Multiple sequence alignments were performed using the ClustalW algorithm (Thompson et al., 1994). The Tamura-Nei model and MEGA11 software were used for the phylogenetic analysis of sequence data (Tamura and Nei, 1993; Tamura et al., 2021).

## Results

### Genome sequencing of PO212, assembly, and general characteristics

The genome of PO212 was sequenced *via* paired-end Illumina MiSeq technology, providing two DNA-seq libraries: one paired-end small fragment library and a second obtained from long-range DNA fragments. The resulting assembly was performed *de novo* and evaluated in terms of N50 and L50 using Quast. Automated assembly and manual sequence verification yielded an estimated PO212 genome size of 29.82 Mb at ~200× coverage. PO212 genome was organized into 65 scaffolds with an N50 scaffold length of 1.88 Mb, comprising the first six longest scaffolds and reaching an N90 scaffold length of 0.38 Mb, corresponding to the first 18 scaffolds (Table 4 and Supplementary Table S1). PO212 genome had a GC content of 49.07%, similar to that of other *Penicillium* genomes. After the manual curation of automated ORF predictions by AUGUSTUS, we determined at least 10,164 ORFs in the PO212 genome.

**Table 4:** Assembly and gene prediction summary of PO212 and S27 genomes.

Strain	Scaffolds	Assembly Size (Mb)	Largest Scaffold (Mb)	N50 (Mb)	BUSCO completeness (%)	Predicted genes
PO212	65	29.82	3.49	1.88	98.5	10,164
S27	414	29.89	1.65	0.42	98.3	10,164

The data presented in the study are deposited in the GenBank repository, accession number JAPDLE000000000.

### Comparative analysis of PO212 assembly and genomic data from *Penicillium rubens* and *Penicillium chrysogenum* strains

To perform a comparison of PO212 with other genomes, we chose those genomes deposited in databases of *Penicillium* species classified as *P. rubens* or *P. chrysogenum*. Given the controversy in the current classification of *P. rubens* and *P. chrysogenum*, we first analyzed the barcode sequences corresponding to *benA*, *caM*, and *RPB2* in these deposited genomes and the newly sequenced PO212 to determine which strains corresponded to each species.

**Figure 1:**
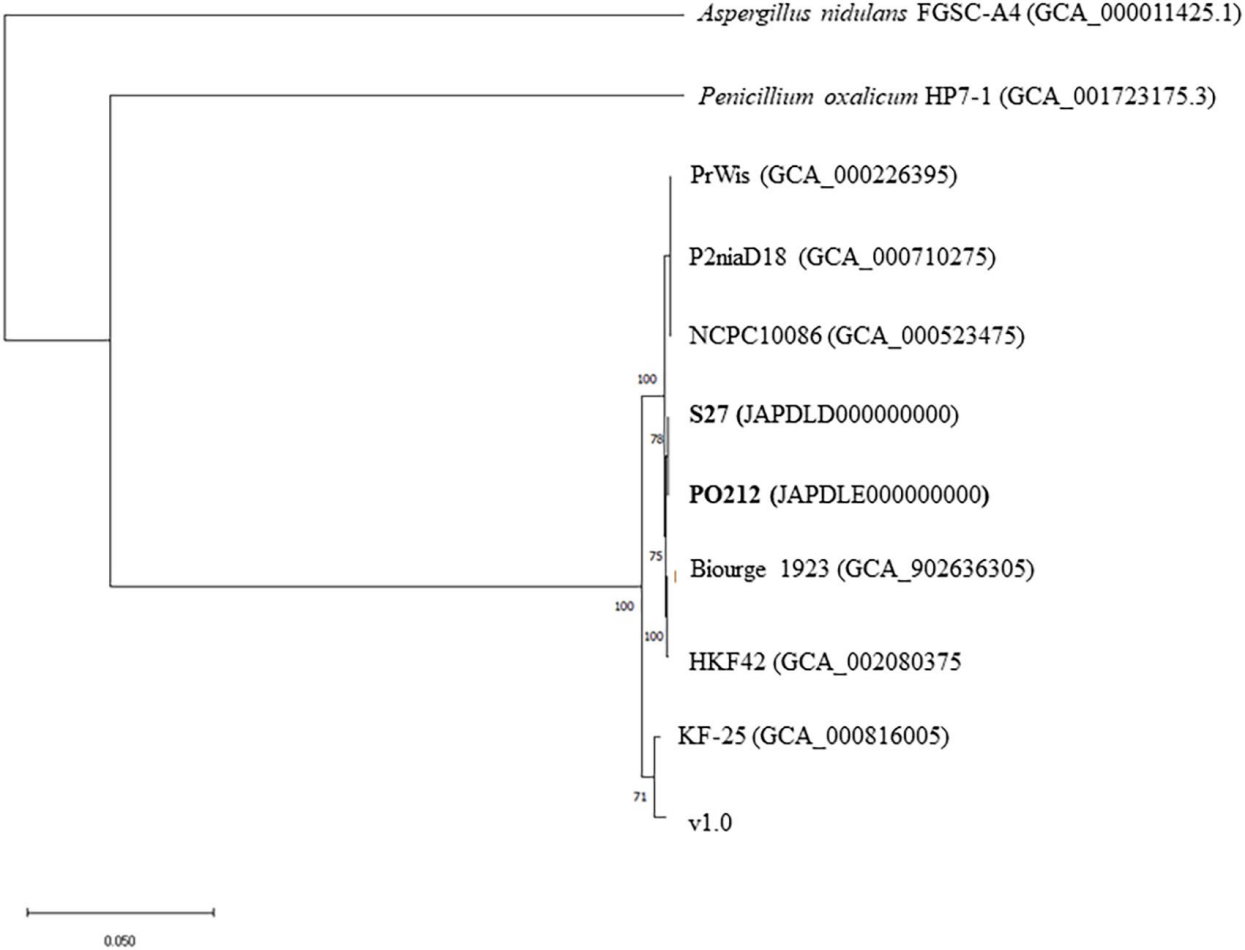
Genome-based phylogenetic tree among *Penicillium rubens* strains studied in this work. The phylogenetic tree was generated using the software JolyTree. The *Aspergillus nidulans* strain FGSC-A4 genome and *Penicillium oxalicum* strain HP7-1 genome were used as outgroups.

Using the Clustal Omega Multiple Sequence Alignment tool (EMBL-EBI), we generated multiple alignments with each of the three barcode sequences (Supplementary Figure S1). These alignments indicated that all strains share the same sequence changes characteristic of *P. rubens* barcodes (indicated as pink boxes in Supplementary Figure S1). In the genomes of strains KF-25 and v1.0, *benA*, *caM*, and *RPB2* barcodes displayed a mixture of sequence changes between those of *P. rubens* and *P. chrysogenum*. This analysis indicated that most probably all strains, including PO212 and with the exception of KF-25 and v1.0, may belong to the *P. rubens* clade, as the nucleotide sequences of the barcodes were identical to those described as *P. rubens* as compared to the sequence described as *P. chrysogenum* (Supplementary Figure S1). Supplementary Figure S2 shows the tree generated for all studied strains of *Penicillium* spp. by concatenating the sequences of *benA, caM*, and *RPB2* gene fragments (between 1,879 and 1,931 bp). This tree clustered the multilocus sequence of *P. rubens* strain DTO 98E8 and all multilocus sequences from previously described *P. rubens* strains and differentiated those from *P. chrysogenum* strains KF-25 and v1.0. This cluster of *P. rubens* strains included PO212 multilocus. Consistent with the multilocus analysis shown in Supplementary Figure S2, a genome-based phylogenetic tree constructed using nucleotide sequence from assemblies of all studied strains showed the identical distribution of strains into two clusters (Figure 1).

For a more detailed comparison, multiple comparative analyses of these genomes were conducted using the Quast tool and the PO212 genome as a reference genome (Supplementary Table S1). The genomes of strains KF-25 and v1.0 were included as distant members of the *P. rubens* and *P. chrysogenum* clades. All genomes used in this comparison contained similar GC content (Supplementary Figure S3). The percentage of the genome represented relative to the PO212 genome ranged from 92.91 (KF-25) to 98.579% (HKF42). The duplication ratio was low (1.001–1.007), indicating high sequence conservation and the absence of major duplication events in the BCA PO212. In terms of mismatches, the greatest differences were found, as expected, with those that presented the greatest differences in barcode alignments (KF-25 and v1.0). However, the most similar strains to PO212 in the number of mismatches were HKF42 and Biourge 1923. The best-assembled genome, organized into five scaffolds, is P2niaD18 and the comparison with PO212 assembly showed the presence of 98.46 mismatches per 100 kbp, probably indicating the distant origins of both strains.

**Figure 2:**
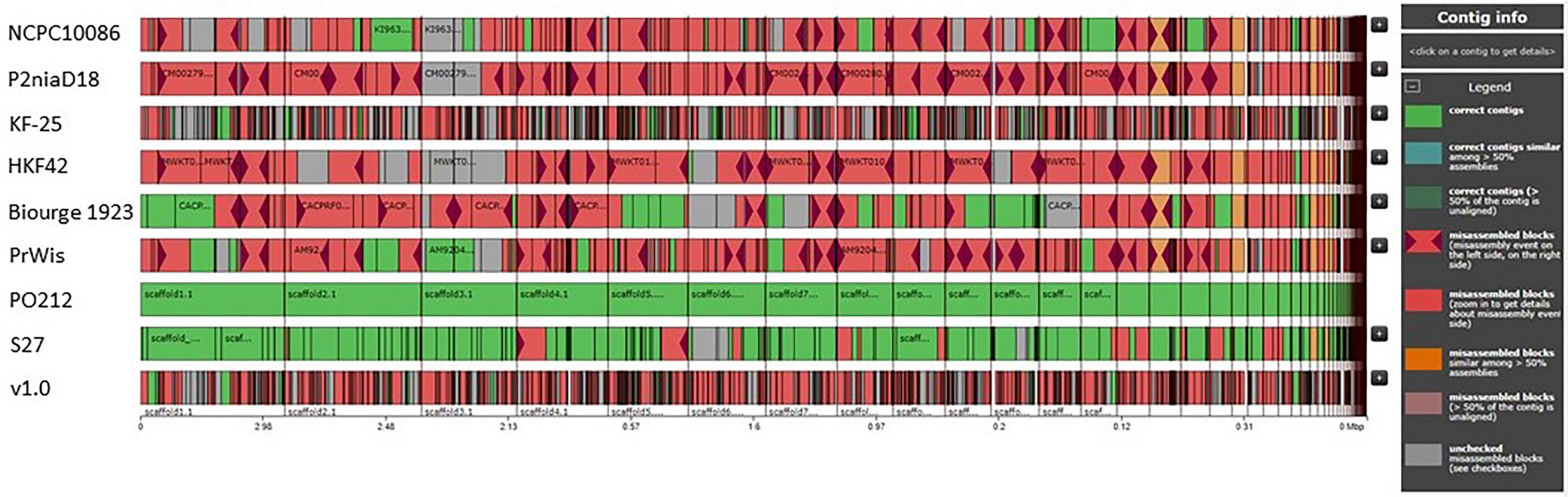
Comparative graphs of P2niaD18, PrWis, Biourge 1923, NCPC10086, KF-25, HKF42, v1.0, S27, and PO212 genomes. Contig alignment viewer, showing the organization of the genomes with reference to PO212. Green regions are right contigs, blue regions are correct contigs similar among >50% assemblies, and in dark green are the contigs referring to correct contigs (>50% of the contigs are unaligned). Pink regions with garnet triangles refer to misassembled blocks (misassemblies events on the left side or the right side), whole pink regions refer to misassembled blocks, orange regions are misassembled blocks (similar among >50% assemblies), brown regions are misassembled blocks (>50% of the contigs are unaligned), and gray regions are unchecked misassembled blocks (Visualized with Icarus).

Quast analysis also showed the differences between the PO212 assembly and the other selected *Penicillium* genomes, suggesting the presence of numerous variations that could prevent immediate identification of those causing the BCA phenotype. Figure 2 provides an overview of the distinctive organization of the assemblies used in this comparison with respect to PO212. The excessive number of variations and the difficulties in testing biocontrol characteristics of the strains with a sequenced genome prompted us to choose for sequencing and detailed genomic analysis an alternative *P. rubens* strain that lacked the BA but was close to the PO212 strain.

### S27 lacks efficacy against Fusarium wilt

**Figure 3:**
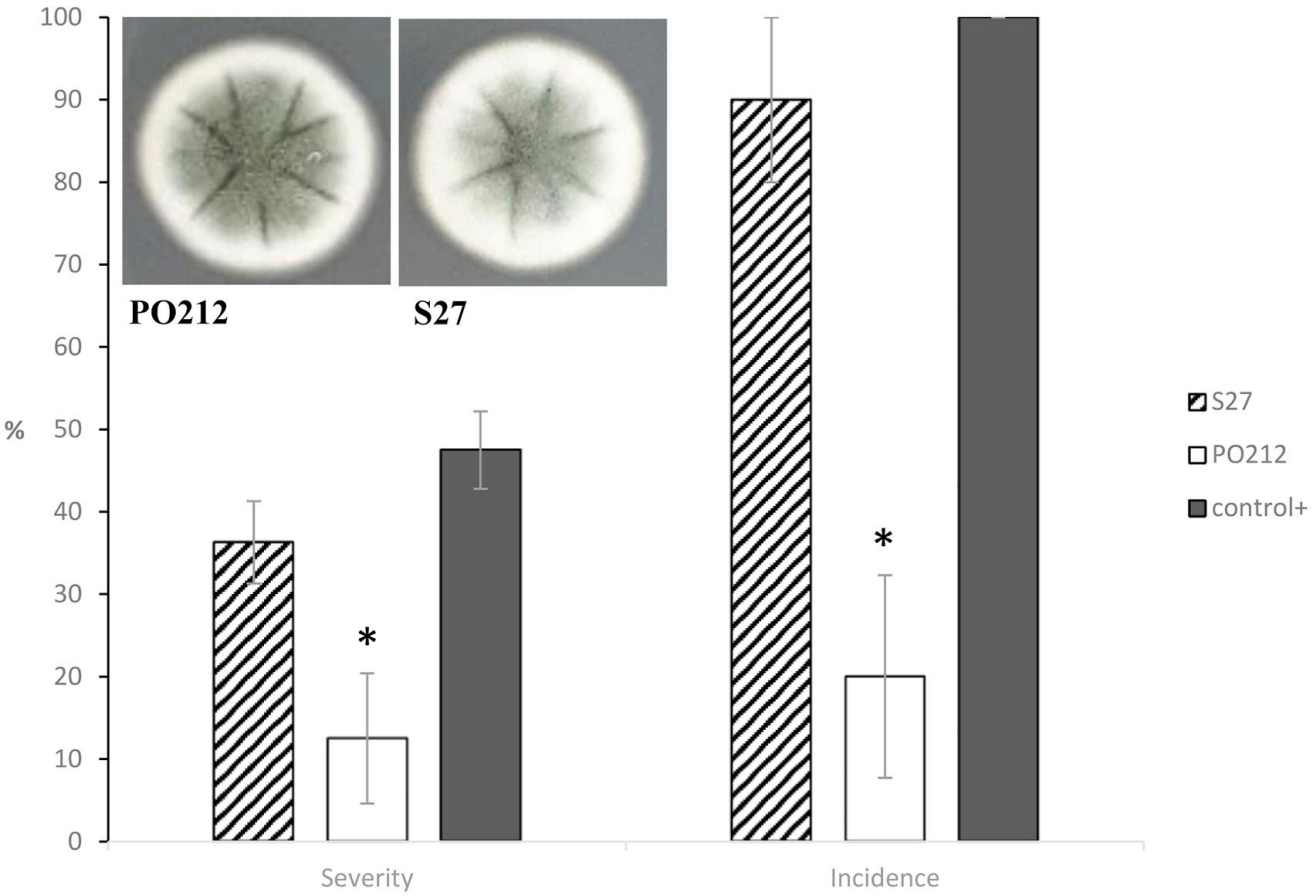
Cultures of 7-day-old *Penicillium rubens* strains 212 and S27 growing on potato dextrose agar (PDA) at 25°C (front). The graph shows the effect of PO212- and S27-treatment on percentage of disease severity and incidence caused by *Fusarium oxysporum* f.sp. *lycopersici* (FOL) in tomato plants cv. “San Pedro” at 28 days after FOL-inoculation under controlled growth chamber conditions. PO212 and S27 treatments were applied to seedlings 7 days before transplanting by watering with a conidial suspension to a final concentration of 6 × 10^6^ conidia g^−1^. Control+, untreated and FOL-inoculated plants. Data are the mean value of five replicates (flasks) per treatment and four plants per replicate. Asterisks in each parameter are significantly different from each other (*p* < 0.05) according to the Student–Newman–Keuls multiple range test. Vertical bars represent the standard error of the mean of five replicates. MSE is the mean squared error of ANOVA. SME 182.292 (Severity) and 416.667 (Incidence).

The S27 strain was isolated from the soil and was primarily classified as a non-BA strain (Villarino et al., 2016). Notably, S27 displayed similar colonial morphology to PO212 (Figure 3). Sequencing of ITS1-5.8S-ITS2 regions classified S27 as a *P. rubens* strain, and a dendrogram based on BOX and repetitive extragenic palindromic (REP) DNA fingerprints placed S27 close to the isolate PO212 (Villarino et al., 2016).

The efficacy of S27 against Fusarium wilt in tomato plants was recently determined. In contrast to PO212, strain S27 did not significantly (*p* ≤ 0.05) reduce either the disease severity or incidence caused by FOL in tomato plants. FOL-inoculated and S27-treated plants showed similar symptoms to FOL-inoculated and untreated control plants (control +, Figure 3). Thus, we chose S27 for genomic sequencing as the best tool for a comparative genomic analysis with PO212 to target genes potentially related to PO212 BA.

### Comparative of PO212 and S27 genomes

The genome of the S27 strain was sequenced using the Illumina Hiseq 4000 platform by 150 bp paired-end sequencing reads that generated 2,345 Mbp (15,532,752 sequence reads). These reads showed coverage of ~82.3× over PO212 assembly. *De novo* assembly of the S27 genome was performed and evaluated in terms of N50 and L50 using Quast. Automated assembly and manual sequence verification allowed estimating for the S27 strain a genome size of 29.89 Mb. S27 genomic assembly was organized into 414 scaffolds with an N50 scaffold length of 0.42 Mb. At least 10,164 coding sequences were estimated after manual curation of ORF prediction by AUGUSTUS.^2^ Barcode analyses of *P. rubens* and *P. chrysogenum* showed that S27 was also a *P. rubens* strain (Supplementary Figure S1). Table 4 shows a summary of the two genomes. The data presented in the study are deposited in the GenBank repository, accession number JAPDLD000000000.

**Figure 4:**
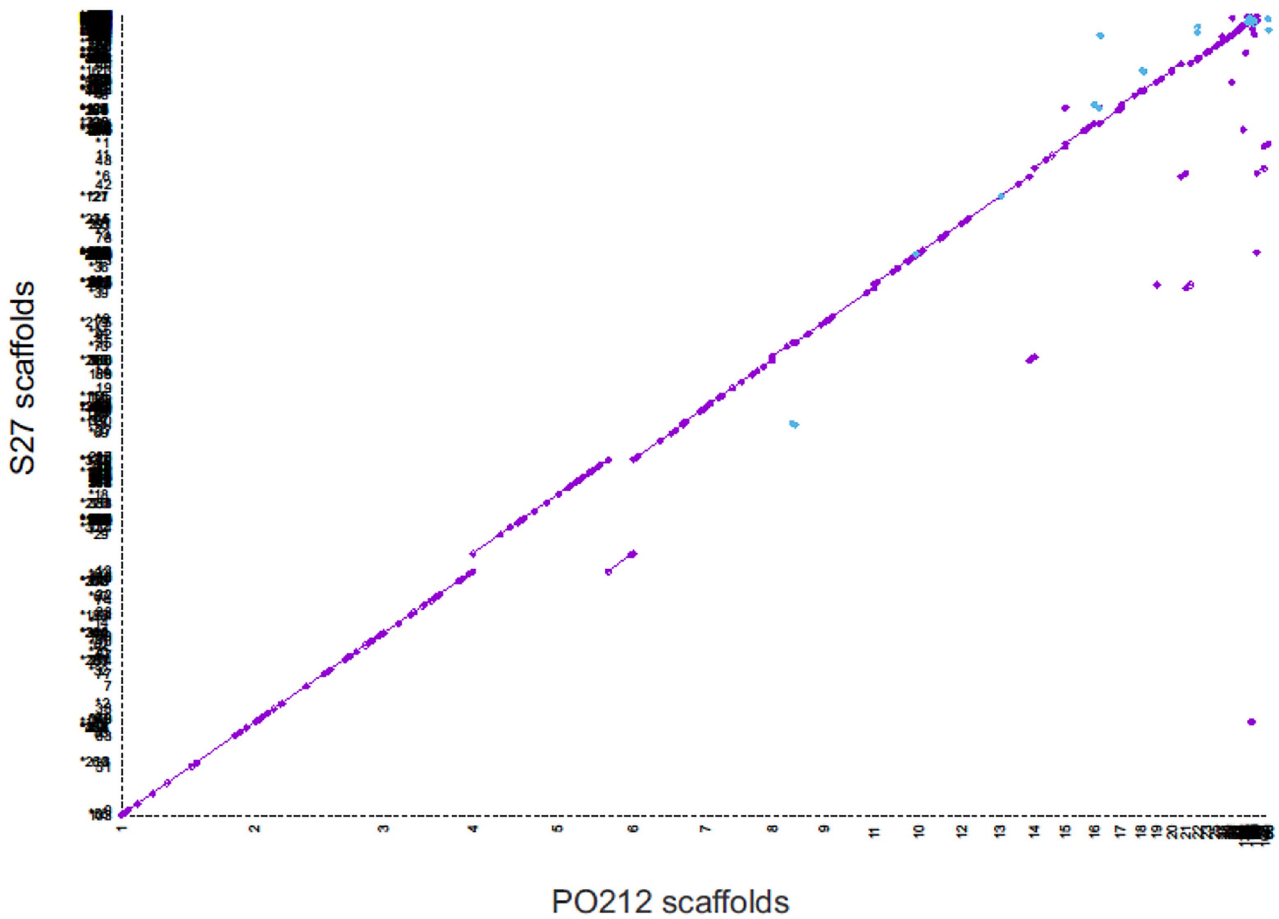
Whole-genome dot-plot showing regions of forward alignment (in purple), translocations (displacements from the diagonal), inversions (in blue), and duplications (parallel diagonal lines) between *Penicillium rubens* strains 212 (PO212) and S27. The PO212 assembly (*X* axis) was used as a reference.

Genome wide alignment showed the strong conservation of PO212 and S27 genomic sequences (Figure 4). MUMmer plot showed that 99.95% of the PO212 genome assembly matched that of the S27 strain with identity equal to or greater than 75%. Only 0.05% of the genomic sequence seemed to be unique to PO212 but did not contain any predicted gene model.

Quast analysis (Supplementary Table S1) also revealed the high similarity between PO212 and S27 genomes. Comparisons of the S27 and PO212 assemblies showed that up to 99.81% of the S27 genome was represented in PO212 and a duplication ratio of 1.002. In this comparison 1,890 mismatches were found, a rate of 6.34 changes per 100 kbp of genomic sequence, which was significantly lower than the previous genomic comparison (Supplementary Table S1). The phylogenetic trees using either multilocus or genomic analysis also confirmed the proximity of the two strains PO212 and S27 and other *P. rubens* strains by joining them in a clade (Figure 1 and Supplementary Figure S2).

**Figure 5:**
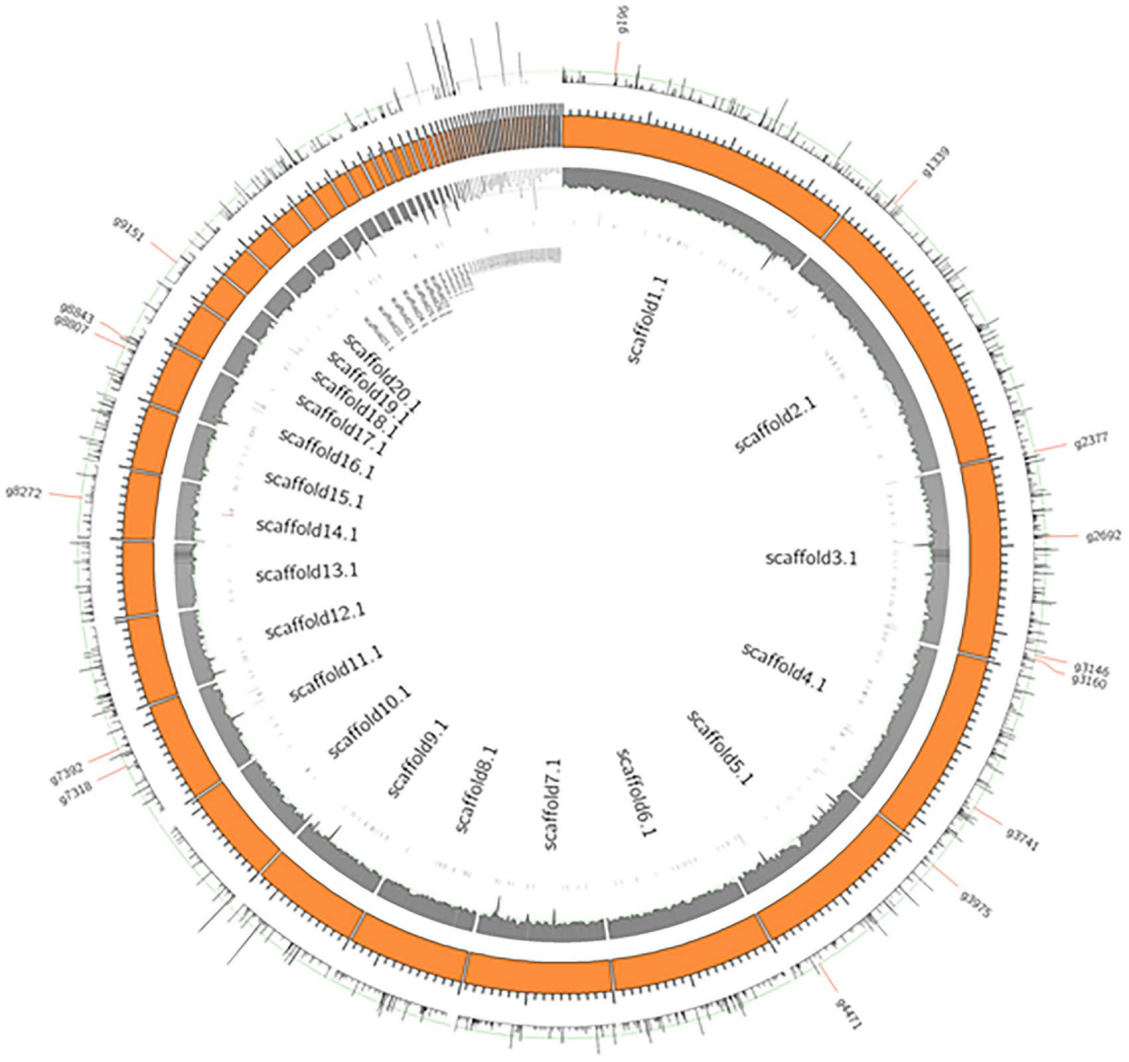
Genome wide SNVs of PO212 genome against S27. Orange boxes present scaffolds of the PO212 genome. The first inner track, the gray histogram, shows the coverage of the S27 reads aligned against the PO212 genome. The data show full coverage of the PO212 genome. The outermost track represents the SNV density of the PO212 genome against the aligned S27 reads. The innermost track, black ticks, represents genes containing SNVs in their coding region. 15 genes are highlighted with longer ticks in the innermost track and labeled in the outermost track. These 15 genes showed 100% allelic variation on the SNV loci of PO212 when compared with S27.

**Figure 6:**
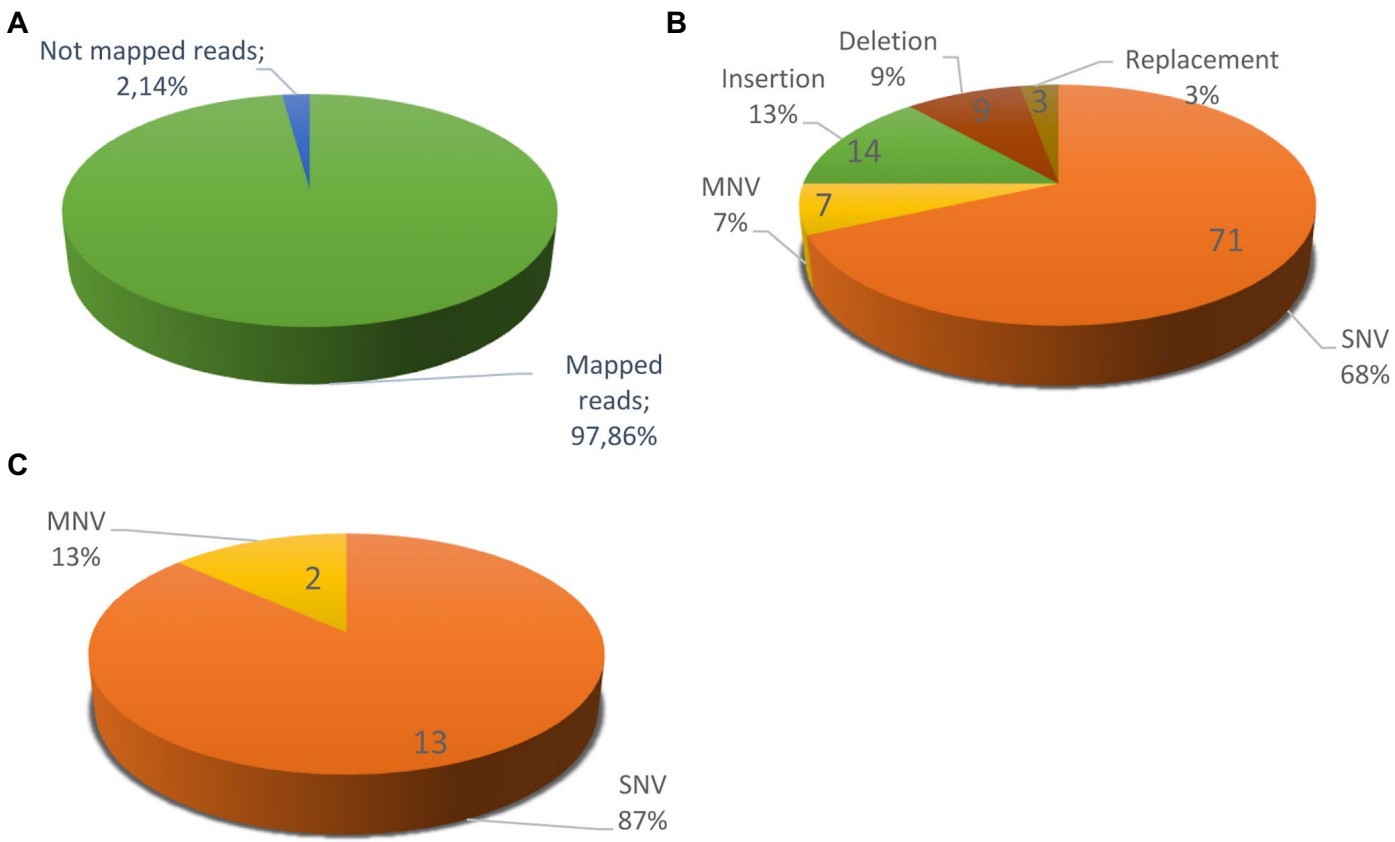
Variations between *Penicillium rubens* strains 212 (PO212) and S27 genomes. **(A)** S27 mapped reads vs. the un-mapped reads using PO212 as a reference genome. **(B)** Differences between two strains classified according the type of mutation: 71 SNVs, 7 MNVs, 14 Insertions, 9 Deletions, and 3 Replacements. **(C)** Differences between the two strains in the coding regions with 100% of frequency: 13 SNVs and 2 MNVs. After a review with preliminary RNA-Seq data support, we discarded seven variations, considered them as mistakes, and classified two as silent variations, since they did not cause an amino acid change (Supplementary Table S2).

**Figure 7:**
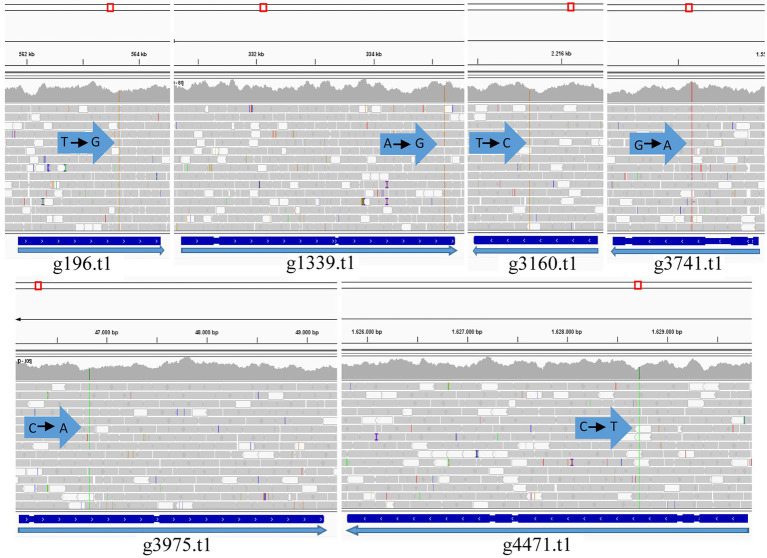
Graph mapping the S27 reads against the PO212 reference genome in the genes containing the variation (PO212 genes): g196.t1 (Dimethylglycine oxidase), g1339.t1 (Vegetative incompatibility protein HET-E-1), g3160.t1 (Putative mitochondrial chaperone), g3741.t1 (Hypothetical protein), g3975.t1 (RNA polymerase I specific TF), and g4471.t1 (Hypothetical protein). Arrows indicate the point of variation and nucleotide change (Visualized with IGV).

Figure 5 shows a circos ideogram representing a comparison between the assembly of PO212 and S27 raw reads. S27 sequencing data present full coverage of the PO212 genome. Figure 6A shows that 97.86% of S27 reads mapped over the 65 scaffolds PO212 genomic assembly. The remaining 2.14% of S27 reads mapped to the mitochondrial genome. With the aid of Circos analysis and CLC software, we searched for those Single Nucleotide Variants (SNVs) displaying 100% allelic variation between S27 and PO212 genome. We detected at least 104 variations between both genomic sequences. Most of this low number of variations was classified as SNVs (Figures 5, 6B). In addition to 71 nucleotide changes found in coding or intergenic regions, we found seven Multi-Nucleotide Variants (MNVs), 14 insertions, nine deletions, and three replacements (Figure 6B). Only 15 of these variations were found in coding regions (Figures 5, 6C). However, manual curation still reduced the number of variations to six with amino acid change (see Figure 7 and Table 5) and two silent mutations (Supplementary Table S2).

**Table 5:** List of the genes whose sequences present variations between PO212 and S27 strains and the affected amino acid.

Coding region change PO212/S27	Scaffold in PO212 genome	Triplet PO212/S27 (5′➔ 3′)	Amino acid change	Protein	*P. rubens* Wisconsin 54-1255
g196.t1/S27g134.t1	1.1	TGC/GGC	Cys605Gly	Dimethylglycine oxidase	Pc13g04270
g1339.t1/S27g7851.t1	2.1	GAT/GGT	Asp1447Gly	Vegetative incompatibility protein HET-E-1	Pc12g06410
g3160.t1/S27g8511.t1	3.1	TCC/CCC	Ser271Pro	Putative mitochondrial chaperone	Pc21g18720
g3741.t1/S27g3030.t1	4.1	GGT/AGT	Gly135Ser	Hypothetical protein	Pc06g00910
g3975.t1/S27g4439.t1	5.1	CCT/CAT	Pro219His	RNA polymerase I specific TF	E8E15_002124
g4471.t1/S27g3229.t1	5.1	CGA/TGA	Arg327^*^	Hypothetical protein	Pc16g08360

We focused on six genes that carried an SNV causing an amino acid change. Only one SNV caused an early stop in the coding sequence. In g4471.t1 gene (orthologue of Pc16g08360 in PrWis), the CGA codon for arginine 327 was changed to a TGA stop codon. This SNV caused the truncation of the hypothetical protein S27g3229.t1 at amino acid 326, missing 3/4 of the ORF compared to the predicted PO212 protein (g4471.t1; Table 5). The remaining SNVs described in Table 5 caused punctual amino acid substitutions (Figure 7). Notably, these SNVs were found only in S27. The orthologues of PO212 showed the same nucleotide sequences as in the PrWis and P2niaD18 reference genomes.

We then confirmed the presence of these variations between S27 and PO212 genomes by PCR amplification of these regions and subsequent sequencing (Table 6). To investigate whether any of these variations were specific to the biocontrol phenotype, we chose eight strains from our collection of *P. rubens* strains classified accordingly to their BA. We sequenced the regions where those SNVs were mapped. We found that SNVs are present in g196.t1, g1339.t1, and g3169.t1 genes (nomenclature as in PO212 genome) specific to S27 (Table 6). For the remaining genes, it was feasible to establish two well-differentiated groups based on specific changes in g3741.t1, g3975.t1, and g4471.t1 (Table 6).

**Table 6:** Nucleotide variations and alleles in loci of *Penicillium rubens* strains from INIA, CSIC collection.

Strain	BCA^a^	MAT alleles	*nirA* alleles	g196.t1	g1339.t1	g3160.t1	g3741.t1	g3975.t1	g4471.t1
PO212	+	MAT1-1	*nirA1*	T	A	T	G	C	C
S27	−	MAT1-1	*nirA1*	G	G	C	A	A	T
S17	−	MAT1-1	*nirA1*	T	A	T	A	A	T
S71	−	MAT1-1	*nirA1*	T	A	T	A	A	T
S73	+	MAT1-1	*nirA1*	T	A	T	A	A	T
CH2	−	MAT1-2	WT	T	A	T	G	C	C
CH5	−	MAT1-2	WT	T	A	T	G	C	C
CH6	+	MAT1-1	*nirA1*	T	A	T	G	C	C
CH8^*^	+	MAT1-1	WT	T	A	T	G	C	C
CH16	+	MAT1-1	*nirA1*	T	A	T	G	C	C

BCA^a^, biological control agent. The symbol + indicates biocontrol activity. The symbol – indicates no biocontrol activity. ^*^CH8 carries the crnA1 mutation. The name of the genes corresponds to PO212.

Given the absence of specific mutations or genes that could explain the BA of PO212, we performed a search for putative homologs of 13 genes involved in biocontrol in *Trichoderma* species (Table 7; Sharma et al., 2011). Twelve were found in the genomes of PO212 and S27 without any difference in nucleotide sequences.

**Table 7:** List of genes described in *Trichoderma* spp. as genes related to biocontrol (Sharma et al., 2011).

Genbank number	Gene	Function	PrWis code	PO212 code	S27 code
AM050097	Squalene epoxidase (*erg1* gene)	Silencing of the *erg1* gene enhances resistance to terbinafine that shows antifungal activity	Pc22g15550	g7259.t1	S27g7219.t1
EU124654	Putative acetyltransferase and monooxygenase	Antagonist activity against *S. sclerotiorum*, *S. minor*, and *S. cepivorum*	Pc21g05060	g8955.t1	S27g5480.t1
EU311400	Heat shock protein 70 kDa (*hsp70* gene)	Increases fungal resistance to heat and abiotic stresses	Pc22g11240	g9809.t1	S27g8859.t1
AJ605116	mRNA for endochitinase (*ech42* gene).	Antifungal activity in transgenic tobacco	Pc13g09520	g648.t1	S27g7976.t1
EF407410	Carotenoid cleavage dioxygenase 1 (*ccd*1 gene)	Helps in hyphal growth, conidiospore development and carotenoid pigment production	Pc12g09530	g1595.t1	S27g829.t1
EU551672	Transcription factor CTF1 (*ctf1* gene)	Antifungal activity against *R. solani*, *Fusarium oxysporum*, and *B. cinerea* and production of 6- pentyl-2H-pyran-2	Pc21g11250	g5805.t1	S27g1152.t1
DQ910533	Protease gene SL41	Biocontrol activity against pathogens	AAG44693	g5366.t1	S27g8455.t1
AM421521	Endopolygalacturonase (*pg1* gene) exons 1–5	Secretion of plant cell wall degrading enzymes against *R. solani* and *P. ultimum*	Pc22g20290	g9752.t1	S27g1693.t1
EU399786	Hypothetical kelch domain containing protein (*Thkel1*)	Expression of this gene in *A. thaliana* modulates glucosidase activity, and enhances tolerance to salt and osmotic stresses	Pc13g04170	g188.t1	S27g126.t1
AY156910	Xylanase (*xyl* gene)	Helps in breakdown of hemicellulose	Pc12g01520	g7689.t1	S27g1384.t1
Accession number not available	*tmkA* gene	Induction of plant systemic resistance and biocontrol activity against *R. solani*. (Tested in green house condition)	Pc22g01670	g7951.t1	S27g4744.t1
Accession number not available	*qid74* gene	Involved in cell protection and adherence to hydrophobic surfaces that helps in antagonism against *R. solani*	not located	not located	not located
Accession number not available	*Sm1* gene	A small cysteine-rich protein that induces defense responses in dicot and monocot plants and in protecting crop diseases	Pc20g15140	g6346.t1	S27g2607.t1

The GenBank number, the gene name, and the function are detailed. In addition, the code in PrWis, PO212, and S27 if the gene is present in these strains.

## Discussion

*Penicillium rubens* strain 212 is a BCA not only capable of reducing the vascular wilt of tomatoes caused by FOL but also other diseases in a variety of horticultural crops (Larena et al., 2003a; De Cal et al., 2008, 2009; Martinez-Beringola et al., 2013). The main mode of action for the control of Fusarium wilt by PO212 is the induction of resistance in tomato plants (De Cal et al., 1997b, 1999, 2000), although plant growth promotion and competition for space and nutrients are also described (De Cal et al., 1995; Sabuquillo et al., 2009). Despite these numerous studies, the molecular basis of PO212 BA remains unexplored. Therefore, the main objective of this study was to understand the genetic components governing the biocontrol mechanism. For this purpose, we sequenced and assembled the PO212 genome for comparison with other *Penicillium* genomes deposited in databases.

A *de novo* assembly of the PO212 genome was performed, yielding a genomic sequence organized into 65 scaffolds. The estimated size of the PO212 genome (29.82 Mb) is consistent with the predicted genome size from other strains such as KF-25 (29.91 Mb; Peng et al., 2014) and Biourge 1923 (30.45 Mb; Pathak et al., 2020). However, other *Penicillium* genomes have a larger genome, as it is the case of P2niaD18 and NCPC10086 with 32.5 and 32.3 Mb, respectively (Specht et al., 2014; Wang et al., 2014).

With the assembled genome of PO212 and the barcodes of the *benA*, *caM*, and *RPB2* genes (Visagie et al., 2014), we confirmed that PO212 belongs to the *P. rubens* clade, as previously described by Villarino et al. (2016). In addition, the PO212 genome was compared with the genomes of other *P. rubens* and *P. chrysogenum* strains to assess the quality of the PO212 assembly. These comparisons pointed to KF-25 and v1.0 as distant relatives of the other *Penicillium* strains, which were easily included in the *P. rubens* clade. *P. chrysogenum* and *P. rubens* are morphologically very similar, making it difficult to classify them into these clades without the aid of molecular data (Houbraken et al., 2011). Genome comparisons using the Quast tool allowed us to establish the strains most similar to PO212, but the lack of knowledge of whether the strains whose genomes are compared are BCAs and the differences between strains do not help us to delve deeper into the genetic basis that governs the biocontrol mechanism. Therefore, we considered the sequencing and comparison of a local strain more similar and geographically close to strain PO212 but lacking BA, so we selected isolate S27 (Villarino et al., 2016).

Similar studies comparing biocontrol microorganisms with non-biocontrol microorganisms of the same species (Hernández-Salmerón et al., 2017) and different species (Kubicek et al., 2011) have found notable differences and thus provide a genetic basis for understanding the biocontrol process. Following that strategy, Wang et al. (2014), through a comparison of the genomes of NCPC10086 and PrWis, identified 69 genes located in different scaffolds of strain NCPC10086. However, comparative analysis of the PO212 and S27 genomes showed strong conservation of their genomic sequences. Only six variants, causing an amino acid change, were found in the coding regions between PO212 and S27. Sequencing of these ORFs in eight other strains from our stock collection, previously classified according to their BA, showed the presence of specific variations in soil isolates and those taken from plants, but no correlation was found with their BAs. We also took advantage of these strains to determine whether the presence of known mutations or variations was the cause of BA. First, we determined the presence of MAT1-1, which is believed to play an important role in several biotechnological traits (Böhm et al., 2013) or MAT1-2. This analysis yielded no correlation between these loci and BA; in fact, both PO212 and S27 carry the MAT1-1 locus. Second, we had previously studied the complementation of the *nirA1* mutation causing a nitrate-assimilation deficient phenotype in PO212 (Espeso et al., 2019). Although complementation of *nirA1* mutation in PO212 transformants caused a reduction in their biocontrol phenotype, the S27 and other non-biocontrol strains carried the same *nirA1* mutation, indicating that the loss of NirA activity has no role in BA. In conclusion, PO212 and S27 are two strains very similar in sequence, with one important difference, the biocontrol capacity of PO212. However, current data do not suggest any correlation between the presence of the SNVs found and the biocontrol capacity of PO212 nor the absence or presence of extra genes specifically related to this phenotype.

Genome comparisons of *Postia placenta* strains evidenced high similarity between their genomes while showing important differences in phenotypes (Kölle et al., 2020). Hence, the high conservation of PO212 and S27 genomic sequences points to the presence of specific variants located in non-coding regions as candidates for a role in biocontrol. When located in putative promoter sequences, these variations could cause changes in gene expression patterns. Transcriptomic analyses are a convenient approach to studying the expression patterns of candidate genes with a potential role in biocontrol as previous works have shown (Jiang et al., 2019; Morán-Diez et al., 2019). Future lines of research will find the basis of the biocontrol phenotype in *Penicillium* focusing on epigenetics or the presence of RNA-based mycoviruses.

## Data availability statement

The datasets presented in this study can be found in online repositories. The names of the repository/repositories and accession number(s) can be found in the article/supplementary material.

## Author contributions

EE and IL conceived and designed the experiments. ER and MV performed the experiments. ER, LA, and JV carried out trimming and mapping of sequencing reads. ER and JV analyzed the data. ER, EE, and IL wrote the original draft manuscript. All authors contributed to the article and approved the submitted version.

## Funding

The work at the INIA-CSIC laboratory was supported by the RTA2013-00060-C05-01 (Plan Nacional de Ministerio de Economía y Competitividad), RTA2017-00019-C03-01 (Plan Nacional de I + D, MICIU, Spain), and PID2021-123594OR-C21 (MCIN/AEI/10.13039/501100011033/FEDER, UE). ER received a scholarship from the MICIU.

## Conflict of interest

The authors declare that the research was conducted in the absence of any commercial or financial relationships that could be construed as a potential conflict of interest.

## Publisher’s note

All claims expressed in this article are solely those of the authors and do not necessarily represent those of their affiliated organizations, or those of the publisher, the editors and the reviewers. Any product that may be evaluated in this article, or claim that may be made by its manufacturer, is not guaranteed or endorsed by the publisher.

## Footnotes

^1^ https://www.bioinformatics.babraham.ac.uk/projects/fastqc/

^2^ http://bioinf.uni-greifswald.de/augustus/
