# Supplementary figures and images for: Comparative analysis of Penicillium genomes reveals the absence of a specific genetic basis for biocontrol in Penicillium rubens strain 212
Source: Front Microbiol. 2023 Jan 13;13:1075327. doi: 10.3389/fmicb.2022.1075327 (PMC9880469; doi:10.3389/fmicb.2022.1075327)

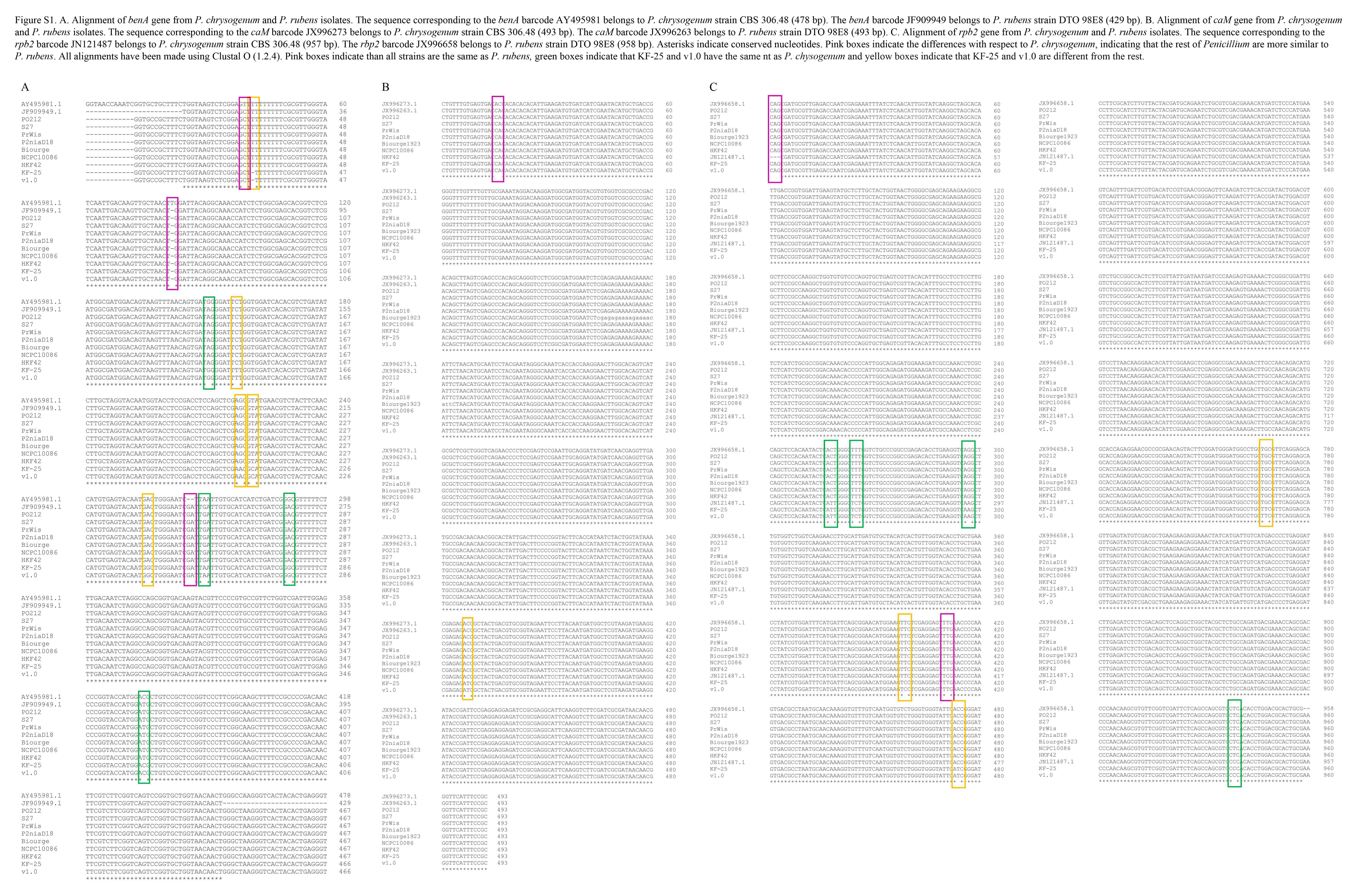

Supplement: Supplementary file 3 [file Image_1.JPEG]

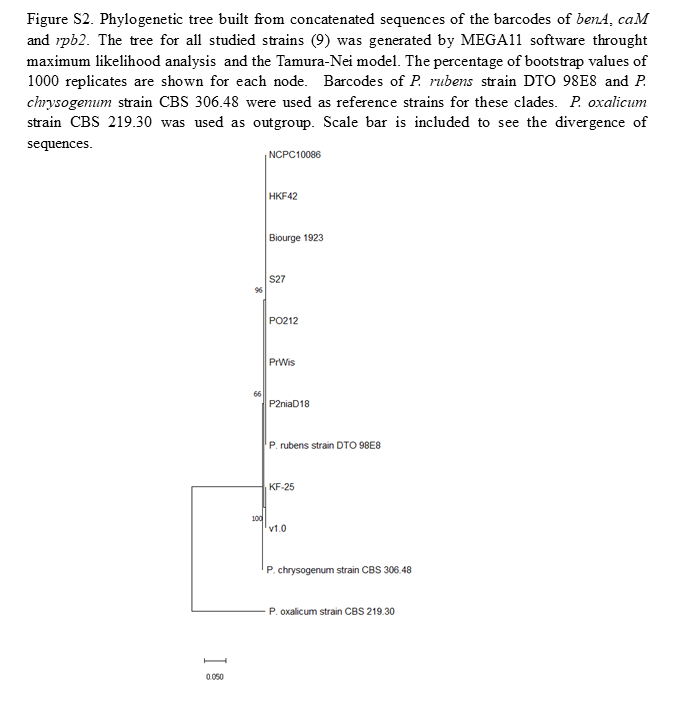

Supplement: Supplementary file 4 [file Image_2.TIF]

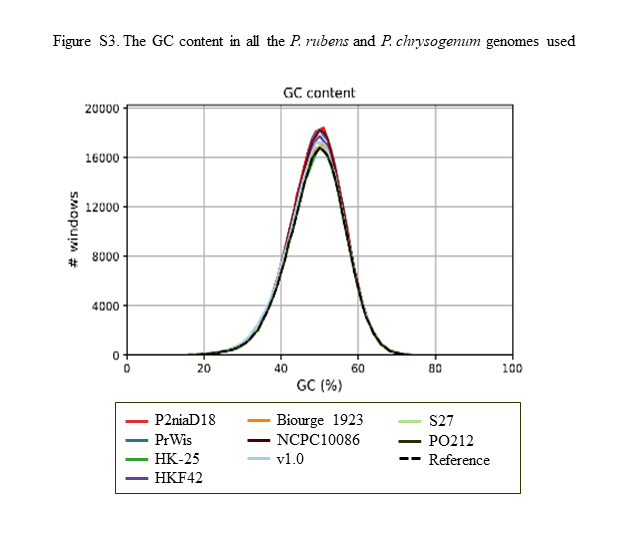

Supplement: Supplementary file 5 [file Image_3.TIF]
